# Halogen‐Mediated Polymer‐Free Recovery of Pristine MoS_2_ Monolayers From Gold‐Assisted Exfoliation

**DOI:** 10.1002/smtd.70913

**Published:** 2026-07-29

**Authors:** Jeyavelan Muthu, Farheen Khurshid, You‐Chen Lin, Ya‐Ping Hsieh, Martin Kalbáč

**Affiliations:** ^1^ Department of Low‐Dimensional Systems Heyrovský Institute of the CAS Prague Czech Republic; ^2^ Institute of Atomic and Molecular Sciences, Academia Sinica Taipei Taiwan; ^3^ Department of Physics National Taiwan University Taipei Taiwan; ^4^ Nano Science and Technology Program Taiwan International Graduate Program, Academia Sinica Taipei Taiwan; ^5^ Molecular Science and Technology Program Taiwan International Graduate Program, Academia Sinica Taipei Taiwan

**Keywords:** bi‐phasic, etching, exfoliation, polymer‐free, TMDs

## Abstract

Metal‐assisted mechanical exfoliation has recently emerged as a powerful strategy for producing large‐area monolayers of two‐dimensional (2D) transition‐metal dichalcogenides (TMDs) with near‐intrinsic crystalline quality. The strong adhesion between the TMD monolayer and the metallic adhesion layer poses a significant challenge for device integration, as it generally requires polymer‐assisted transfer processes that introduce contamination, mechanical strain, and structural defects. Here we report a halogen‐mediated biphasic etching strategy that enables the polymer‐free recovery of TMD monolayers directly from gold‐assisted exfoliation substrates. Sequential iodine vapor exposure followed by liquid‐phase iodine etching weakens the Au‐TMD interfacial interaction and subsequently removes the gold adhesion layer while preserving the structural integrity of the monolayer. Spectroscopic characterization, time‐resolved morphological analysis, and first‐principles calculations reveal that iodine adsorption modifies the electronic structure of the Au surface and reduces its binding strength to the TMD layer. MoS_2_ monolayers recovered using this approach exhibit significantly reduced strain, lower charge doping, and suppressed wrinkle formation compared with polymer‐transferred counterparts. Optoelectronic devices fabricated from these monolayers demonstrate enhanced photocurrent and improved operational stability. These findings establish halogen‐mediated biphasic etching as a scalable route for integrating pristine 2D semiconductors with technologically relevant substrates and provide a promising pathway toward high‐performance 2D optoelectronic devices.

## Graphical abstract

Schematic procedure for halogen‐mediated polymer‐free recovery of pristine MoS_2_ monolayers from gold‐assisted exfoliation.

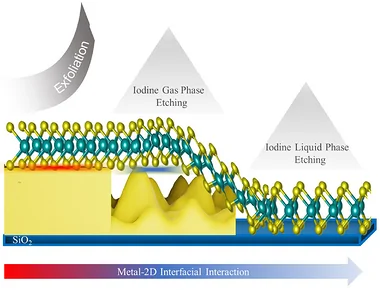

## 1 Introduction

Two‐dimensional (2D) semiconductors have attracted substantial attention owing to their unique electronic structure, strong light–matter interactions, and potential for next‐generation optoelectronic technologies [1, 2]. Among these materials, transition‐metal dichalcogenides (TMDs) such as MoS_2_ exhibit direct band gaps at the monolayer limit and possess exceptional mechanical flexibility and chemical stability, making them promising candidates for applications including photodetectors, transistors, and flexible electronic systems [3, 4, 5, 6, 7].

A central challenge in this field is the scalable production and integration of high‐quality monolayers on technologically relevant substrates. Mechanical exfoliation remains the benchmark method for obtaining pristine 2D crystals, however, it typically yields small flakes with limited monolayer coverage [8, 9]. Alternative approaches, such as chemical vapor deposition [10], enable the synthesis of large‐area films but frequently introduce grain boundaries [11, 12], point defects [13, 14], and chemical contamination [10, 15] that degrade device performance.

Metal‐assisted mechanical exfoliation (MME) has recently emerged as an effective strategy for producing large‐area monolayers with near‐intrinsic crystal quality [16, 17, 18]. In this approach, a metal film, most commonly gold, is used to promote exfoliation through strong interfacial interactions between the metal surface and the chalcogen atoms of layered materials. These interactions enable the isolation of millimeter‐scale monolayers with high yield [16, 19]. However, the strong Au‐TMD adhesion [20] that facilitates exfoliation also presents a major obstacle for device fabrication, as the metallic adhesion layer must be removed without damaging or contaminating the monolayer.

Existing approaches typically rely on polymer‐assisted transfer processes to detach the monolayer from the metal substrate [16, 21, 22]. Although effective, these processes often introduce polymer residues [23, 24], interfacial contamination [25, 26], and mechanical strain during transfer [27], which can significantly degrade the intrinsic optical and electronic properties of the material.

Addressing this challenge requires strategies capable of selectively weakening the metal–TMD interaction while preserving the structural integrity of the atomically thin crystal. In this work, we introduce a halogen‐mediated biphasic etching strategy that enables the polymer‐free recovery of TMD monolayers directly from gold‐assisted exfoliation substrates. In this approach, iodine vapor first modifies the Au surface and weakens the Au–TMD interaction, after which a liquid‐phase iodine etchant removes the gold adhesion layer. Combined experimental characterization and first‐principles calculations reveal the interfacial mechanism underlying this process. Using MoS_2_ as a model system, we demonstrate that the recovered monolayers exhibit minimal strain, reduced charge doping, and improved surface morphology relative to polymer‐transferred materials. Devices fabricated from these monolayers show enhanced optoelectronic performance, highlighting the importance of interface cleanliness in 2D semiconductor systems.

## 2 Results and Discussion

Compared to graphene, TMDs exfoliated on Au exhibit a much stronger adhesion (∼1.207 J m^−2^) [28], making conventional polymer‐free transfer approaches (Table S3), such as bubbling [29], surface‐tension processes [30], Laser‐induced dry ablation [31], and tape stamping [32] energetically lessfavorable.

**FIGURE 1:**
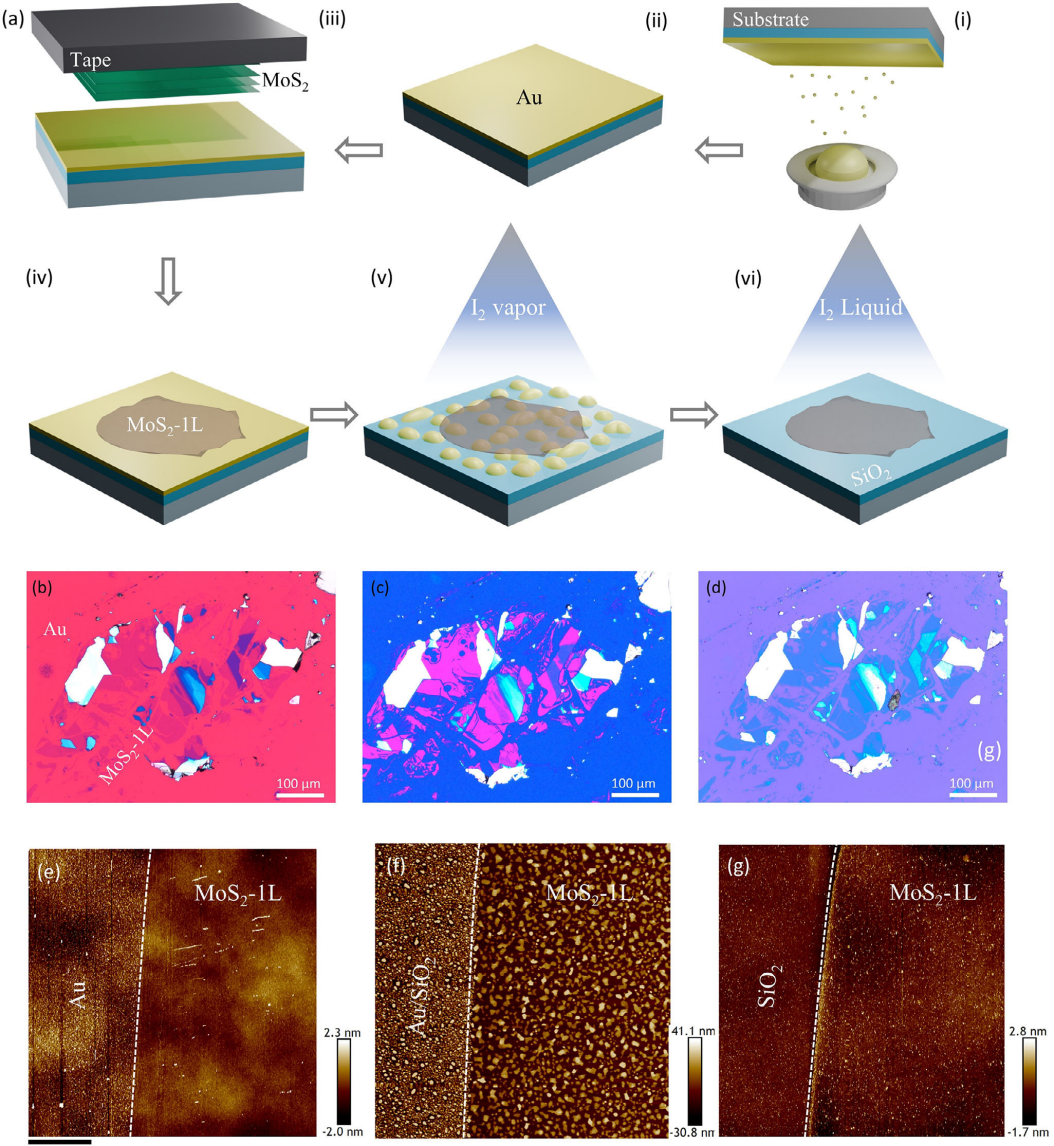
Biphasic etching protocol and the morphological evolution of MoS_2_. (a) Schematic illustration of the biphasic iodine etching strategy for polymer‐free recovery of monolayer MoS_2_. (i) Sputter deposition of an Au film onto a Si/SiO_2_ substrate. (ii) Au‐coated Si/SiO_2_ substrate used for metal‐assisted mechanical exfoliation. (iii) Freshly cleaved bulk MoS_2_ on adhesive tape brought into contact with Au‐coated SiO_2_ substrate. (iv) Large‐area monolayer MoS_2_ exfoliated onto the Au‐coated substrate. (v) Iodine vapor‐phase etching partially removes the Au beneath the MoS_2_, reducing the metal–MoS_2_ interfacial contact while establishing localized adhesion to the SiO_2_ substrate. (vi) Subsequent KI/I_2_ liquid‐phase etching completely removes the residual Au, yielding a pristine monolayer MoS_2_ directly supported on the SiO_2_ substrate. (b–d) Optical microscopy (OM) images showing the corresponding morphological evolution of MoS_2_ during the etching process. (e–g) Atomic force microscopy (AFM) images of MoS_2_ before etching and after vapor‐ and liquid‐phase etching. scale bar: 5 µm.

To illustrate this issue, we exfoliated MoS_2_ monolayer onto Au (Figure 1a(i–iv)) [16, 20, 33, 34]. For this purpose, a freshly cleaved MoS_2_ bulk crystal was brought into contact with an Au‐coated SiO_2_ substrate. Subsequent peeling resulted in large‐area monolayers (lateral size > 100 µm) along with a small fraction of few layer/bulk flakes on the substrate. In contrast, direct exfoliation onto SiO_2_ without an Au adhesion layer typically produced much smaller monolayer flakes with lateral size below 10 µm (Figure S1).

We developed a two‐step iodine etching process to selectively etch the buried Au layer. First, a gas‐phase etching protocol (Figure 1a(v)) using sublimated iodine (I_2_) was employed to weaken the interaction between the Au layer and MoS_2_. The etching process was conducted in a custom‐fabricated vacuum‐sealed reaction cell (Figure S2), where samples were exposed to I_2_ vapor generated from solid iodine pellets at 125°C. Upon exposure, the I_2_ vapor diffuses beneath the loosely bound edges of the 2D layer and initiates etching of the underlying Au. Control experiments on Au films of varying thicknesses (Figure S3) revealed that gas‐phase iodine etching effectively disrupts Au films thinner than 20 nm, fragmenting the continuous Au layer into isolated Au islands. Therefore, a subsequent liquid‐phase iodine etching step (Figure 1a(vi)) was carried out to completely remove the residual Au, while preserving the integrity of the MoS_2_ monolayer.

Optical microscopy after etching reveals a striking enhancement in the visibility of MoS_2_ monolayers due to a change in optical contrast from selective Au removal (Figure 1b–d). Atomic force microscopy (AFM) was employed to investigate the topographical evolution of MoS_2_ after the etching process. The as‐exfoliated MoS_2_ on a continuous Au film (Figure 1e) exhibited a uniform, smooth surface with the root‐mean‐square (RMS) surface roughness measured to be around 0.17 nm, indicating minimal topographical variation [35]. Following gas‐phase etching with sublimated iodine, the roughness significantly increases (Figure 1f), reaching a surface roughness of ∼6.49 nm. We hypothesize that the Au layer beneath the MoS_2_ was disrupted, forming discontinuous gold nanostructures, the detailed mechanism is discussed in a later section.

Following this initial gas‐phase etching step, complete removal of the residual Au was achieved using a potassium iodide/iodine (KI/I_2_)–based liquid etching solution. The Au dissolution process during the gas‐and liquid‐phase etching steps can be represented by the following reactions:

$$2\mathrm{Au}\left(s\right)+I_{2}\left(g\right)\to 2\mathrm{Au}I\left(s\right)$$

$$2\mathrm{Au}I\left(s\right)+2I^{-}\left(\mathrm{aq}\right)\to 2{\left[\mathrm{Au}I_{2}\right]}^{-}\left(\mathrm{aq}\right)$$

Notably, this biphasic etching process enables a complete restoration of the MoS_2_ onto the dielectric, preserving flake morphology as obtained during exfoliation. Such full‐coverage restoration is not achievable with conventional polymer‐assisted transfer methods, which frequently suffer from incomplete transfer, tearing, residual strain or structural distortion during the release and redeposition steps [23, 36, 37].

AFM upon completion of the liquid‐phase KI/I_2_ etching step confirms that the Au layer was completely removed, leaving the MoS_2_ in direct contact with the underlying SiO_2_ substrate (Figure 1g). The extracted surface roughness is around 0.23 nm, approaching that of the pristine state. Importantly, this post‐etching topography (Figure S4) was devoid of the high‐density wrinkles or ripples commonly observed in polymer‐assisted transfer methods [25, 26, 38]. A line scan (Figure S4d) was performed on the biphasic‐etched sample, and the step height was measured to be ∼0.8 nm [39], corresponding to the expected thickness of a monolayer and indicating the complete removal of residual Au and Iodine.

The post‐etched MoS_2_ was further examined by X‐ray photoelectron spectroscopy (XPS). The survey spectrum (Figure S5) shows no detectable Au or I signal, indicating a complete removal of the Au adhesive layer and the absence of residual Iodine species after KI/I_2_ etching. These results confirm that the biphasic etching process restores MoS_2_ without leaving metallic or etchant‐derived contaminants.

To verify the critical role of the iodine vapor step, freshly exfoliated MoS_2_ was directly immersed in KI/I_2_ solution without prior vapor etching. Under these conditions, the MoS_2_ flakes were completely detached from the substrate (Figure S6), demonstrating that the vapor‐phase etching is essential for establishing partial contact between MoS_2_ and the SiO_2_ substrate. This partial anchoring provides sufficient mechanical support during the subsequent liquid‐phase etching, enabling complete Au removal while preserving the integrity of the monolayer.

Although the biphasic etching process involves sequential vapor‐ and liquid‐phase etching, both steps are simple, solution‐compatible, and readily scalable. Unlike conventional polymer‐assisted transfer, our approach is performed entirely on the original substrate without mechanical delamination, polymer coating/removal, or post‐transfer thermal annealing.

**FIGURE 2:**
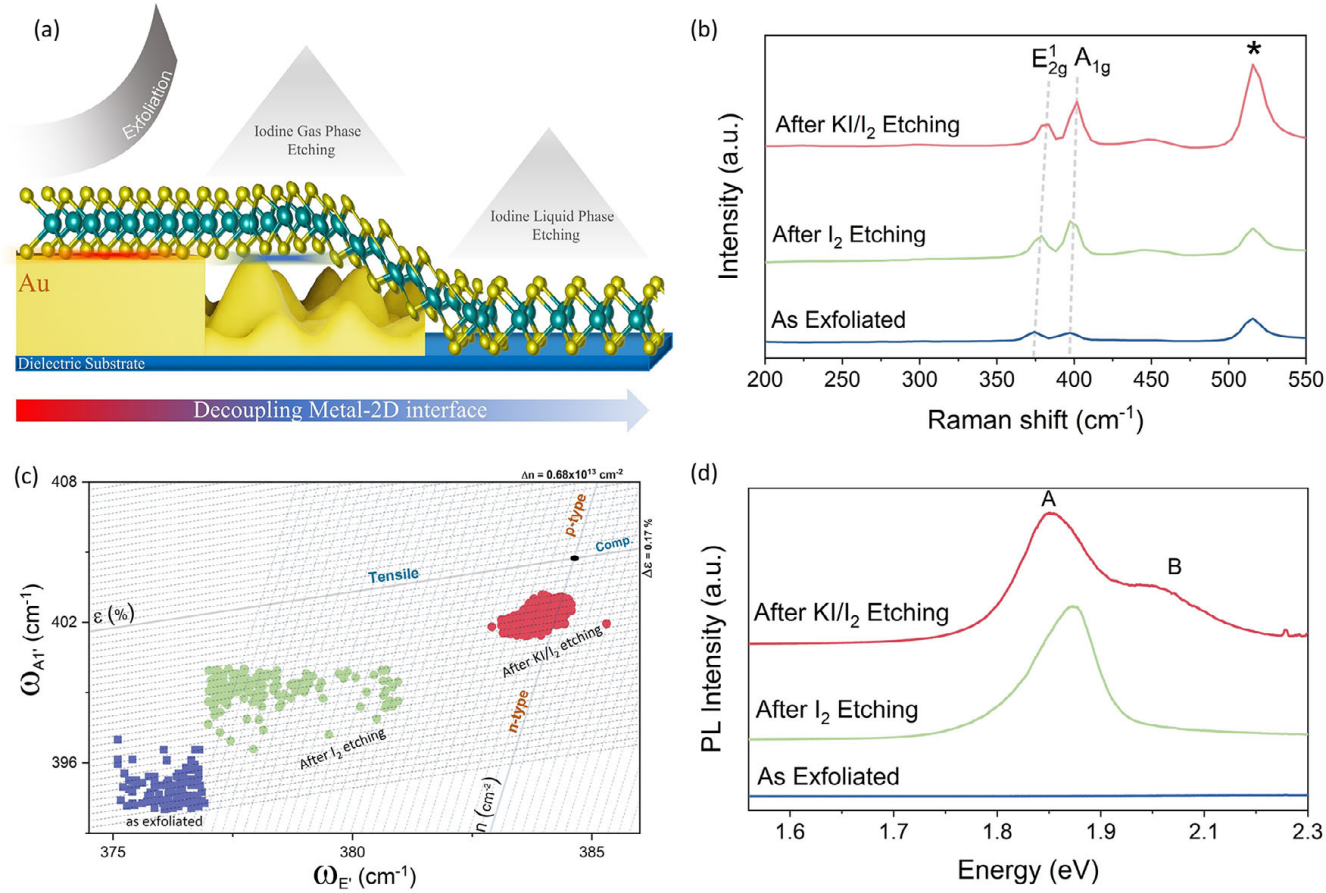
Structural and optical evolution of MoS_2_ during the biphasic iodine etching process. (a) Schematic illustration of the biphasic etching Process. (b) Micro‐Raman spectra of MoS_2_ before and after biphasic etching; the peak at 520 cm^−1^ (marked by an asterisk) corresponds to the Si substrate. (c) Statistical strain–doping correlation analysis derived from the relative shifts of the E_2g_ and A_1g_ modes. (d) Photoluminescence (PL) spectra showing the progressive decoupling of MoS_2_ from Au and restoration of its intrinsic excitonic emission following biphasic etching.

Figure 2a schematically illustrates the morphological evolution of MoS_2_ during the different stages of the biphasic etching process. The corresponding structural evolution was monitored by micro‐Raman spectroscopy (Figure 2b). For the as‐exfoliated MoS_2_ on Au, the E_2g_ and A_1g_ vibrational modes were significantly red‐shifted relative to those of monolayer MoS_2_ on SiO_2_, indicating a pronounced phonon softening arising from Au‐MoS_2_ interfacial coupling and charge transfer [34, 40]. Upon iodine vapor exposure, both Raman modes progressively blue‐shifted, reflecting the weakening of the metal‐2D interaction as the continuous Au film became fragmented beneath the MoS_2_. Following complete Au removal by liquid‐phase etching, the E_2g_ and A_1g_ modes further shifted toward ∼384 and ∼403 cm^−1^, respectively, closely matching the characteristic frequencies of monolayer MoS_2_ directly exfoliated onto SiO_2_ [41]. This spectral evolution indicates the progressive relaxation of the interfacial tensile strain together with suppression of charge transfer.

We quantify the evolution of doping and strain during the biphasic etching process through statistical correlation analysis (Figure 2c) from the spatial Raman map (Figure S7). The as‐exfoliated MoS_2_ exhibited pronounced n‐type doping and significant tensile strain, consistent with charge transfer from the underlying Au substrate as well as mechanical stress imparted by the adhesive tape during exfoliation [42]. Upon iodine vapor exposure, both the doping level and tensile strain were partially alleviated which supports the proposed reduction of Au‐MoS_2_ interfacial coupling as iodine disrupts continuous metal contact and suppresses interfacial charge transfer. Following the subsequent KI/I_2_ liquid‐phase etching step, the residual Au was completely removed, further decreasing the doping level and shifting the strain state toward a slightly compressive regime. This progression highlights the effectiveness of the biphasic etching process in not only decoupling MoS_2_ from metallic substrates but also in restoring its intrinsic morphology by mitigating extrinsic strain and charge transfer effects.

To verify this result, we conducted photoluminescence (PL) spectroscopy (Figure 2d) before and after biphasic etching. For the as‐exfoliated MoS_2_ on Au, no measurable PL emission was observed due to the strong interfacial charge transfer and nonradiative recombination pathways at the Au‐MoS_2_ interface [43]. After iodine vapor exposure, a distinct PL peak emerged that indicates the suppression of interfacial interaction. The peak is centered around 1.87 eV, corresponding to the A exciton transition. Upon complete Au dissolution by liquid etching, both the A (1.86 eV) and B (2.01 eV) excitonic transitions are clearly resolved, in agreement with the intrinsic excitonic spectrum of MoS_2_ on SiO_2_ [44]. An exciton exhibits a slight redshift relative to the partially etched state, which is likely associated with the transition from partial Au support to direct contact with the SiO_2_ substrate. The resulting change in the dielectric environment slightly alters the exciton binding energy [45]. The recovery of both A and B excitonic features confirms that the biphasic etching process effectively restores the intrinsic optoelectronic properties of MoS_2_.

**FIGURE 3:**
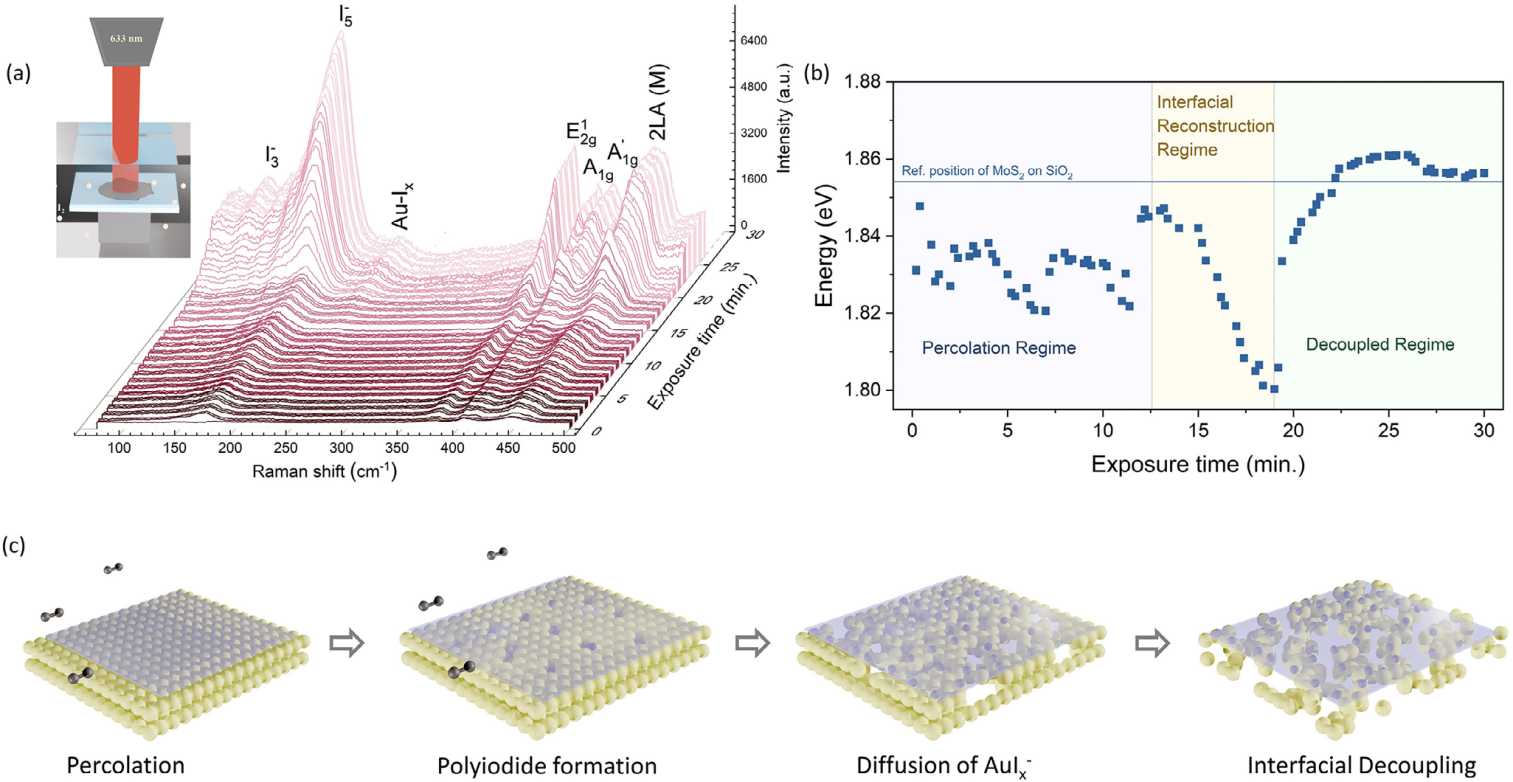
Au‐MoS_2_ decoupling mechanism. (a) In situ Raman under 633 nm excitation and (b) photoluminescence (PL) evolution dynamics (under 633 nm excitation) of MoS_2_‐Au heterointerface during I_2_ vapor‐phase etching. (c) Schematic illustration of the progressive MoS_2_‐Au decoupling process.

The onset and progressive decoupling of the MoS_2_‐Au heterointerface was probed by in‐situ Raman spectroscopy under 633 nm (Figure S8) and 532 nm (Figure S9) excitation. Figure 3 displays the real‐time chemical and electronic evolution of the MoS_2_/Au heterointerface during exposure to I_2_ vapor. The Raman spectra (Figure 3a) provide direct evidence for iodine activation at the interface. Upon exposure, a set of low‐frequency modes emerges at approximately110, and 172 cm^−1^, together with a band near 224 cm^−1^. These modes are absent prior to iodine exposure and are not intrinsic phonons of 2H–MoS_2_ but are instead consistent with the formation of iodine‐derived polyiodides (I_3_^−^/I_5_^−^) and metal–iodine bonding (Au–I_x_) [46, 47, 48], indicating that iodine first percolates and reacts primarily with the Au surface and the MoS_2_/Au interface. With increasing exposure time, the intensities of iodine and MoS_2_‐related modes grow markedly, reflecting the diffusion and accumulation of Au–I_x_ species at the interface.

This process progressively disrupts the continuity of the metal contact, leading to spatially discontinuous Au regions that can support localized plasmonic enhancement. Notably, the MoS_2_ phonon region undergoes a pronounced change after approximately20 min of exposure, the out‐of‐plane A_1g_ mode splits into two components (A_1g_ and A_1g_′). Because the A_1g_ phonon mode is highly sensitive to vertical boundary conditions and substrate coupling strength, this splitting provides clear spectroscopic evidence for the emergence of heterogeneous interfacial environments resulting from MoS_2_‐Au decoupling.

The PL evolution (Figure 3b) mirrors this interfacial reconstruction and can be divided into three regimes. In the early stage (0–13 min), the PL peak position remains nearly unchanged, consistent with an incubation period in which iodine is primarily consumed by reaction with Au and formation of iodine/polyiodide species, while MoS_2_ remains largely metal‐coupled. In the intermediate stage (13–19 min), the PL peak red shifts approximately linearly. This red shift is attributed to a reduction of the optical gap dominated by interfacial reconstruction, including tensile strain induced by Au roughening/etching [49] and bandgap renormalization arising from evolving dielectric screening and interfacial dipole formation as Au–I and polyiodide species accumulate [50, 51].

Finally, in the late stage (19–30 min), the PL peak reverses trend and blue shifts toward the reference emission energy (∼ 1.855 eV) [52] of monolayer MoS_2_ on SiO_2_. This recovery reflects the progressive loss of direct MoS_2–_Au contact, suppression of metal‐induced screening and nonradiative pathways, consistent with our density functional tight binding (DFTB) calculations (Figure S10). The coupled Raman and PL dynamics support a mechanism (Figure 3c) in which iodine vapor first percolates and reacts with Au to generate Au–I/polyiodide species, then drives a transition to a spatially heterogeneous and partially decoupled interface, and ultimately yields a largely decoupled MoS_2_ configuration whose optical response approaches that of dielectric‐supported monolayer MoS_2_. While iodine‐derived species remain stabilized at the interface in the vapor‐treated state, subsequent exposure to a KI/I_2_ solution dissolves the polyiodides and residual Au–I_x_ complexes, effectively removing iodine‐derived interfacial products.

**FIGURE 4:**
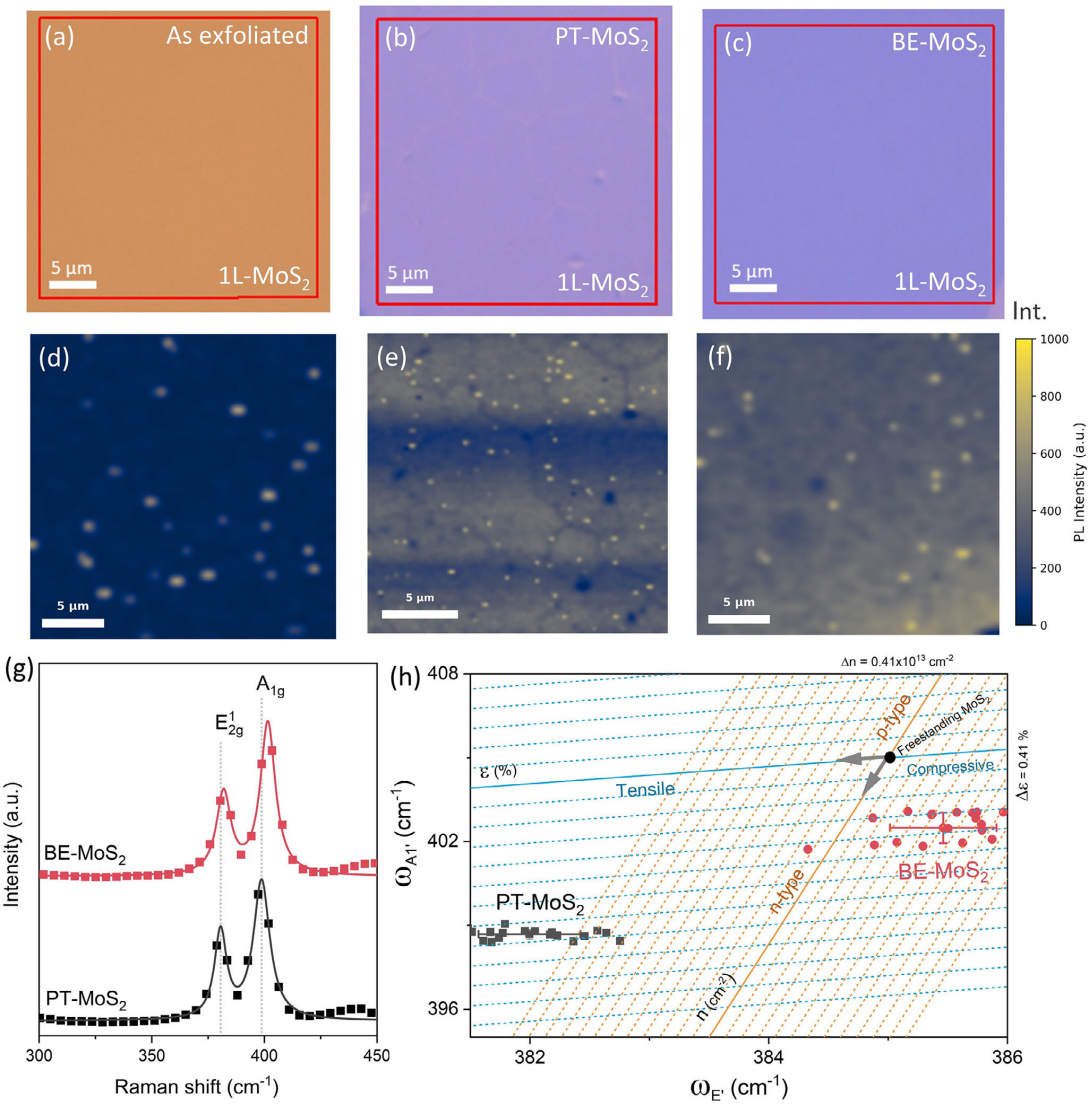
Comparison of biphasic etching with the conventional polymer‐transfer method. (a) High‐resolution optical image of as‐exfoliated MoS_2_ on Au, (b) MoS_2_ transferred onto SiO_2_ by the polymer‐assisted method, and (c) MoS_2_ restored on SiO_2_ via biphasic etching. Corresponding PL maps of (d) as‐exfoliated, (e) polymer‐transferred, and (f) biphasic‐etched MoS_2_, showing differences in emission uniformity. (g) Raman spectra of MoS_2_ prepared by both methods, and (h) strain‐doping correlation plots highlighting the distinct strain and doping states associated with polymer transfer and biphasic etching.

To evaluate the effectiveness of the biphasic etching strategy against conventional polymer‐assisted transfer, we compared polymer‐transferred MoS_2_ (PT‐MoS_2_) with biphasic‐treated MoS_2_ (BE‐MoS_2_). Optical microscope images in Figure 4 show distinct MoS_2_ for both cases. The as‐exfoliated MoS_2_ on Au exhibits a smooth and homogeneous surface, indicative of the pristine nature of the exfoliated flake (Figure 4a). In contrast, polymer‐transferred MoS_2_ (PT‐MoS_2_) shows a discontinuous morphology with visible cracks and surface irregularities (Figure 4b), indicative of mechanical stress introduced during the transfer process [53]. By comparison, biphasic‐etched MoS_2_ (BE‐MoS_2_) displays a smooth and continuous morphology without observable structural damage (Figure 4c), closely resembling the as‐exfoliated state.

We further assess the surface quality and optical uniformity of the resulting MoS_2_ layers by spatially resolved PL mapping under 532 nm excitation, focusing on the A‐exciton emission. The as‐exfoliated MoS_2_ on Au exhibited no detectable PL signal (Figure 4d), consistent with quenching by interfacial charge transfer. However, both PT‐MoS_2_ and BE‐MoS_2_ exhibit a strong PL signal (A‐exciton), signaling restored excitonic properties.

The polymer transferred PT‐MoS_2_ displayed discontinuous PL emission due to transfer‐induced cracks and defects (Figure 4e). Our biphasic BE‐MoS_2_, however, shows a spatially homogeneous emission which highlights the quality of the obtained materials (Figure 4f). Moreover, MoS_2_ transferred using PMMA typically exhibits significant out‐of‐plane deformation due to mechanical strain [53], often necessitating high‐temperature annealing [26] or chemical treatments [54] to restore surface quality. In contrast, our biphasic method yielded 2D layers with minimal topographical distortion without requiring either high‐temperature annealing or chemical treatments, highlighting its advantage in preserving the structural quality of monolayers during and after exfoliation.

The Raman spectra of PT‐MoS2 and BE‐MoS_2_ (Figure 4g) show that the E_2g_ and A_1g_ modes of BE‐MoS_2_ are blue‐shifted relative to those of PT‐MoS_2_. Furthermore, spatial Raman mapping of PT‐MoS_2_ (Figure S11) reveals an inhomogeneous intensity distribution of the E_2g_ and A_1g_ modes, indicative of mechanically induced surface damage during the polymer transfer process. The strain‐doping correlation analysis (Figure 4h) further reveals that PT‐MoS_2_ retains significant tensile strain, consistent with the previous reports of polymer‐transferred CVD‐grown [55] and mechanically exfoliated MoS_2_ [35]. In contrast, BE‐MoS_2_ remained close to the strain‐free regime, indicating that the biphasic etching process effectively minimizes lattice distortion by enabling direct adhesion of the 2D crystal to the dielectric substrate. The slight variation in the strain–doping correlation of BE‐MoS_2_ relative to Figure 2c is attributed to sample‐to‐sample variations, together with slight differences in the etching conditions. Nevertheless, both datasets consistently demonstrate the reduction of residual strain and doping following biphasic etching.

**FIGURE 5:**
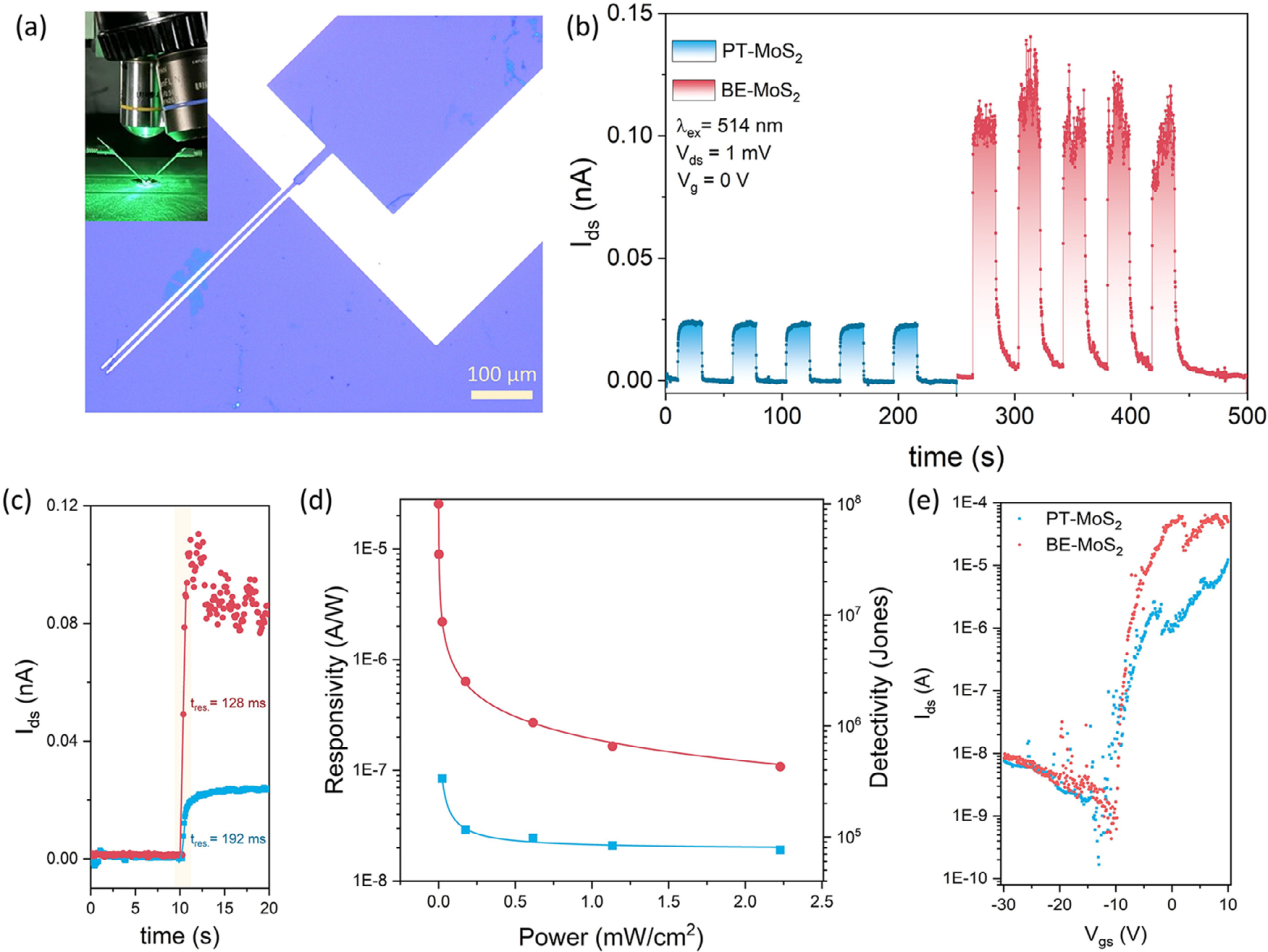
Comparison of device performance for polymer‐transferred (PT‐MoS_2_) and biphasic‐etched (BE‐MoS_2_) photodetectors. (a) Optical image of lithographically patterned source and drain electrodes on BE‐MoS_2_ (inset: photographic image of laser illumination on the MoS_2_ photodetector). (b) Comparative transient photocurrent responses of PT‐MoS_2_ and BE‐MoS_2_ under 514 nm illumination at a bias voltage of 1 mV. (c) Photodetector response times of PT‐MoS_2_ and BE‐MoS_2_, highlighting the faster switching dynamics of BE‐MoS_2_. (d) Power‐dependent responsivity and detectivity of PT‐MoS_2_ and BE‐MoS_2_ devices. (e) Back‐gated transfer characteristics of both PT‐MoS_2_ and BE‐MoS_2_ measured at room temperature with a drain‐source bias of V_ds_ = 2 V.

To study the impact of the improved MoS_2_ quality on its optoelectronic performance, photosensor devices were fabricated from both PT‐MoS_2_ and BE‐MoS_2_. The source and drain electrodes (width = 4 µm) were defined by photolithography followed by thermal evaporation of Cr/Au contacts (Figure 5a). The transient photocurrent response of both devices was measured under 514 nm laser illumination with an external bias of 1 mV (Figure 5b). The slight photocurrent fluctuations observed for BE‐MoS_2_ under illumination. We thank the reviewer for this valuable observation. Although the precise origin of the slight photocurrent fluctuations cannot be conclusively determined from the present measurements, the device exhibits reproducible ON/OFF switching, indicating that these fluctuations do not compromise the operational stability of the photodetector.

Under identical conditions, the photocurrent (I_ph_ = I_light_‐I_dark_) of BE‐MoS_2_ was approximately three times higher than that of PT‐MoS_2_. In addition, BE‐MoS_2_ exhibited a faster response time of 128 ms (Figure 5c). The superior performance of BE‐MoS_2_ can be attributed to its clean interface with the dielectric substrate and the absence of polymer residues, which otherwise act as charge trap sites in PT‐MoS_2_. Residual PMMA and temperature‐induced wrinkles and cracks from the polymer transfer process introduce localized defects and surface roughness, leading to enhanced nonradiative recombination and reduced carrier mobility [56, 57, 58].

Due to the improved on/off ratio even at low excitation power (Figure S12), fivefold enhanced responsivity (Figure 5d) and short response time of BE‐MoS_2_ devices (128 ms), our device exhibits one of the highest reported detectivity values for MoS_2_ devices (Table S2). Furthermore, back‐gated field effect transfer characteristics of the devices were examined (Figure 5e). Minor current fluctuation observed in the ON state, which are likely associated with measurements performed under ambient conditions that can influence the electrical characteristics of back‐gated MoS_2_ field‐effect transistors. The field‐effect mobility was extracted from the linear regime of the transfer characteristics, while the threshold voltage was determined by linear extrapolation. BE‐MoS_2_ exhibited a higher ambient field‐effect mobility of ∼11.8 cm^2^ V^−1^ s^−1^, compared to ∼1.35 cm^2^ V^−1^ s^−1^ for PT‐MoS_2_, accompanied by a less negative threshold voltage (∼ −10.2 V vs. ∼ −12.6 V). These findings demonstrate that polymer‐free fabrication is critical for realizing efficient charge transport, establishing biphasic etching as an enabling strategy for high‐performance 2D optoelectronic devices.

## 3 Conclusions

In summary, we demonstrated a scalable and polymer‐free strategy for fabricating pristine 2D transition metal dichalcogenides (TMDs) using a biphasic iodine etching process that circumvents the limitations of polymer‐assisted transfer. In this approach, iodine vapor initiates partial etching of the Au underlayer, which significantly weakens the metal‐2D adhesion, as confirmed by DFT. A subsequent KI/I_2_ liquid‐phase etching step completes Au removal, enabling direct adhesion of the 2D layer to the dielectric substrate without introducing mechanical damage or chemical contamination. Structural and optical characterizations confirmed that the restored monolayers retain their intrinsic nature, displaying minimal roughness and a substantial reduction in wrinkles relative to polymer‐transferred counterparts. Leveraging this advance, photodetectors fabricated from biphasic‐treated MoS_2_ exhibited significantly enhanced performance compared to devices prepared by traditional polymer‐based transfer. Future studies will focus on extending the biphasic iodine etching strategy to other metal‐assisted exfoliated 2D materials to further establish its general applicability. Overall, this work establishes biphasic iodine etching as a robust and versatile route for the integration of high‐quality 2D materials into next‐generation electronic and optoelectronic devices.

## 4 Experimental Section

### 4.1 MME

Gold films were deposited onto pre‐cleaned Si substrates with a 300 nm SiO_2_ overlayer by magnetron sputtering (Quorum). The substrates were cleaned by O_2_ plasma followed by a chemical cleaning protocol prior to deposition. Freshly cleaved MoS_2_ flakes, obtained from bulk crystals using adhesive tape, were immediately brought into contact with the Au‐coated substrate and gently pressed for ∼2 s. Upon release, the tape transferred large‐area MoS_2_ flakes comprising monolayers, bilayers, multilayers, and bulk regions onto the Au film. The monolayer yield was ∼95% when freshly prepared Au substrates were used, but decreased to <50% if Au was exposed to air for more than 2 min. The highest yield was obtained on 10 nm Au films; thinner films (<10 nm) can further improve exfoliation efficiency as reported previously, but require adhesion layers (Ti or Cr) for stability. Since these adhesion layers exhibit poor reactivity toward iodine etching, they were avoided in this study to ensure efficient post‐exfoliation cleaning.

### 4.2 Iodine Biphasic Etching

Iodine vapor‐phase etching was carried out in a custom‐built vacuum reactor (Figure S1). Iodine crystals (>99%, Merck) were sublimed at 125°C to generate vapor, and the exfoliated samples were exposed to the vapor phase. A color change from yellowish (Au) to bluish (SiO_2_) indicated the onset of etching within a few seconds. Following gas‐phase exposure, samples were rinsed sequentially with deionized (DI) water and acetone to remove residual iodine. The etching rate was governed by iodine concentration and exposure time; higher values accelerated etching but induced redeposition of Au due to iodine condensation inside the chamber. To minimize redeposition, residual iodine vapor was continuously evacuated by a vacuum pump and cooled in a condenser. Exposure conditions were optimized to avoid redeposition artifacts. Gas‐phase etching led to only partial removal of Au beneath MoS_2_, as etching terminated upon the formation of AuI_x_ deposits that blocked further reaction. Complete removal of the residual Au was carried out using a commercially available KI/I_2_‐based gold etchant (Sigma‐Aldrich). To moderate the etching rate and improve process control, the commercial etchant was diluted with deionized (DI) water at a 1:1 (v/v) ratio prior to use. The samples were then immersed in the freshly prepared etchant. Although dilution reduced the etching rate, it increased the etching time from several minutes to a few hours. To compensate for the slower etching kinetics, the liquid‐phase etching was performed at 40°C. The required etching duration depended on both the lateral size of the 2D crystal and the thickness of the underlying Au film. Following KI/I_2_ etching, the samples were rinsed several times with DI water to remove residual Iodine species, followed by sequential rinses with acetone, and isopropanol. The substrates were then dried under a gentle nitrogen flow while maintained at a slight inclination to promote uniform solvent drainage and minimize the capillary forces that could induce wrinkles or bubble formation. No additional thermal annealing was required after the biphasic etching process.

### 4.3 In‐Situ Raman Measurements

In situ Raman measurements were carried out using a 633 nm excitation laser. For iodine exposure, the MoS_2_/Au sample was placed inside a sealed quartz glass chamber, into which iodine vapor was introduced. After sealing, the sample was continuously exposed to iodine vapor at room temperature, and Raman spectra were collected sequentially as a function of exposure time. The reaction kinetics were monitored by tracking the time‐dependent evolution of characteristic Raman and PL features.

### 4.4 DFTB Calculations

DFTB calculations were performed with DFTB+ software package and GFN1‐xTB method (details provided in the Supplementary Information). The adsorption energy of MoS_2_ on Au is calculated using “$E\_\mathrm{adsorption}=E\_\mathrm{system}-\left(E\_\mathrm{Mo}S_{2}+E\_\mathrm{Au}\right)$”.

### 4.5 Optical, Structural, and Morphological Characterizations

AFM was performed using a Bruker Dimension Icon, and surface roughness was analyzed using Gwyddion software. Raman and PL spectra were collected at room temperature on a WITec Alpha 300R microscope under 532 nm excitation, with powers of 1 mW (Raman) and 100 µW (PL). Optical microscopy images were obtained using an Olympus microscope.

### 4.6 Photodetector Fabrication and Characterization

For device fabrication, biphasic‐etched MoS_2_ monolayers (BE‐MoS_2_) on SiO_2_/Si substrates were patterned by photolithography, followed by deposition of Cr/Au (5 nm/70 nm) source and drain electrodes via thermal evaporation. For comparison, MoS_2_ was first exfoliated onto Au‐coated SiO_2_ substrates using the same MME process. The exfoliated MoS_2_ was subsequently transferred onto SiO_2_ using a conventional PMMA‐assisted transfer method to fabricate PT‐MoS_2_. Briefly, PMMA was spin‐coated on MoS_2_/Au, followed by etching of Au in KI/I_2_ solution for 10 h and subsequent transfer of MoS_2_/PMMA onto SiO_2_/Si. PMMA was removed by acetone wash. The active channel length and width of both devices (BE‐MoS_2_ and PT‐MoS_2_) were approximately 8 µm and 65 ± 3 µm, respectively. Back‐gated FET transfer characteristics (V_ds_ = 2 V) and photo response measurements were performed at room temperature (25°C) using a Keithley 2612B dual‐channel source meter. The field effect mobility (µ_FE_) was extracted from the linear regime of transfer characteristics according to

$$\;\mu_{FE}=\frac{L}{WC_{ox}V_{ds}}\;\left(\frac{dI_{ds}}{dV_{g}}\right)$$

Where L and W are the channel length and width, respectively, C_ox_ is the gate capacitance per unit area of the 300 nm SiO_2_ dielectric (11.5 nF cm^−2^), and V_ds_ is the drain‐source bias. The threshold voltage (V_th_) was determined by the linear extrapolation of the transfer curve in the linear regime. For photocurrent measurements, samples were illuminated by a 514 nm laser (HORIBA Jobin‐Yvon Raman system, 100× objective). Transient photocurrent responses were recorded under an external bias of 1 mV, and responsivity was evaluated as a function of excitation power.

## Conflicts of Interest

The authors declare no competing financial interest.

## Supporting information




**Supporting File**: smtd70913‐sup‐0001‐SuppMat.docx.

## Data Availability

The data that support the findings of this study are available from the corresponding author upon reasonable request.
