# Supplementary material for: Halogen‐Mediated Polymer‐Free Recovery of Pristine MoS2 Monolayers From Gold‐Assisted Exfoliation
Source: Small Methods. 2026 Jul 29;10(17):e70913. doi: 10.1002/smtd.70913 (PMC13555654; doi:10.1002/smtd.70913)
Supplement: Supplementary file 1 [file SMTD-10-e70913-s001.docx]

**Supporting Information**

**Halogen-Mediated Polymer-free Recovery of Pristine MoS_2_ Monolayers from Gold-Assisted Exfoliation**

*Jeyavelan Muthu, Farheen Khurshid, You-Chen Lin, Ya-Ping Hsieh, Martin Kalbáč^*^*

J. M., F. K., M. K.

Department of Low-Dimensional Systems, J. Heyrovsky Institute of Physical Chemistry, Prague, 18200, Czech Republic

E-mail: [martin.kalbac@jh-inst.cas.cz](mailto:martin.kalbac@jh-inst.cas.cz)

Y.-C. L., Y.-P. H.

Institute of Atomic and Molecular Sciences, Academia Sinica, Taipei, 10617, Taiwan

Y.-C. L.

Department of Physics, National Taiwan University, Taipei, 10617, Taiwan

Y.-C. L.

Nano Science and Technology Program, Taiwan International Graduate Program, Academia Sinica, Taipei, 10617, Taiwan

Y.-P. H.

Molecular Science and Technology Program, Taiwan International Graduate Program, Academia Sinica, Taipei 10617

**1. Mechanical Exfoliation of MoS_2_**

**Figure S1**. Optical images of the exfoliated MoS2 (a) without and (b) with Au adhesion layer.

**2. Iodine (I_2_) Gas Phase Etching System**

**Figure S2**. (a) Photographic image of the custom-built iodine (I_2_) vapor-phase etching setup, consisting of a reaction cell, hot plate, cooling system, and vacuum pump, all housed inside a fume hood. (b) Optical image of a MoS_2_ sample after iodine vapor etching. (c) Comparison of MoS_2_ samples before and after iodine vapor exposure, where the observed color change from yellowish to bluish reflects the morphological evolution induced by partial Au etching.

**3. Influence of Au Thickness on the I_2_ Etching Dynamics**

**Figure S3.** (a-c) Optical images of MoS_2_ monolayers exfoliated onto Au films with varying thicknesses (10, 20, and 30 nm). (d-f) Corresponding optical morphologies after iodine vapor-phase etching, showing more pronounced etching and clearer SiO_2_ contrast for thinner Au films (10 nm) compared to thicker layers. (g-i) Raman spectral evolution of MoS_2_ monolayers on different Au thicknesses before and after vapor-phase etching, highlighting the emergence of enhanced E_2g_ and A_1g_ peak intensities and slight blue shifts due to modified substrate interactions and partial decoupling from the Au underlayer.

To investigate the influence of Au thickness on the etching dynamics, MoS_2_ was exfoliated onto Au films of 10, 20, and 30 nm (Figure S3a-c) and subsequently exposed to I_2_ vapor for 2 min. In all cases, a distinct optical contrast was observed after exposure (Figure S3d-f), indicative of partial Au removal beneath the MoS_2_. Notably, for the 10 nm Au film, the etching was significantly more effective, as evidenced by the clear visibility of the underlying SiO_2_ substrate, reflecting a substantial reduction in Au coverage. The enhanced color contrast arises from the formation of discontinuous Au nanostructures, which promote strong plasmonic interactions with incident light. This effect is further corroborated by the pronounced Raman signals, where both the E_2g_ and A_1g_ modes exhibited markedly higher intensities compared to the as-exfoliated state (Figure S3g&h). In addition, a slight blue shift of these modes was detected, consistent with modified substrate interactions following partial Au removal. In contrast, MoS_2_ exfoliated onto thicker Au film (30 nm) displayed minimal spectral change, with Raman peak positions and intensities remaining nearly identical to the pristine state (Figure S3i), suggesting that the limited etching time was insufficient to effectively disrupt the thicker Au layers. These results highlight the critical role of Au film thickness in dictating the efficiency of iodine vapor etching and the subsequent optical and vibrational response of MoS_2_.

**4. Iodine Biphasic Etching and Morphological Evolution**

**Figure S4.** (a) Schematic illustration of the biphasic iodine etching process. (b) Optical microscopy images showing the corresponding morphological evolution of MoS_2_ during etching. (c) Optical microscopy image of a post-etched MoS_2_ monolayer on a SiO_2_ substrate and (d) the corresponding AFM morphology (inset: height profile confirming a monolayer thickness of ∼0.8 nm).

**Figure S5.** (a) X-ray photoelectron spectroscopy of post-etched MoS_2_ sample (a) survey spectra and the high resolution (b) Mo 3d and (c) S2p spectra.

**5. Critical Role of Iodine (I_2_) Vapor Etching in the Biphasic Process**

**Figure S6.** (a) Schematic illustration of direct liquid-phase etching, in which the as-exfoliated MoS_2_ sample was exposed to KI/I_2_ solution without prior iodine vapor etching. Optical microscopy images of MoS_2_ (b) before and (c) after direct KI/I_2_ liquid-phase etching, showing complete removal of flakes due to the absence of substrate support during the rapid etching process.

**6. Iodine Biphasic Etching and Structural Evolution**

**Figure S7.** Structural integrity of MoS_2_ before and after biphasic etching. (a) High-resolution optical image of MoS_2_ on Au with the corresponding Raman intensity maps of the (b) E_2g_ and (c) A_1g_ modes. (d) Optical image of MoS_2_ after gas phase etching with the corresponding spatial Raman intensity maps of the (e) E_2g_ and (f) A_1g_ vibrational modes. (g) Optical image of MoS_2_ after liquid phase etching with the corresponding Raman maps of the (h) E_2g_ and (i) A_1g_ modes. The rectangular region denotes the area where Raman mapping was performed.

**7. Morphological and Spectral Evolution of MoS_2_ During I_2_ Etching**

**Figure S8.** (a) Schematic illustration of the in-situ Raman under 633 nm excitation. (b) Evolution of Raman and PL spectra of MoS_2_/Au heterointerface during iodine vapor exposure.

**Figure S9**. (a) Time-resolved high-magnification optical microscopy images showing the morphological evolution of a MoS_2_ monolayer during iodine vapor exposure (0–12 min). The red square indicates the region used for estimating the Au coverage. (b) Corresponding Raman spectral evolution of MoS_2_ as a function of exposure time. (c) Quantitative reduction of Au coverage extracted from optical images as a function of iodine exposure time.

To elucidate the morphological and spectral evolution of monolayer MoS_2_ during iodine vapor exposure, we conducted time-resolved optical microscopy and Raman spectroscopy (under 532 nm excitation) over an exposure window of 0-12 min. As shown in Figure S9a, after 2 min of exposure, distinct morphological changes emerged, with fractured Au domains appearing beneath the MoS_2_, consistent with localized I_2_-induced etching. With increasing exposure time, the density of residual Au decreased progressively, reflecting a gradual reduction in metal coverage. The corresponding Raman spectra (Figure S9b) reveal a pronounced increase in peak intensity accompanied by a blue shift of both the E_2g_ and A_1g_ modes after 2 min exposure. Continued exposure further enhanced these blue shifts, signifying modified interfacial interactions and strain evolution as the MoS_2_ transitioned from Au-supported to SiO_2_-supported regions. The concurrent appearance of a pronounced Si Raman band further corroborates the increasing dielectric contact. Quantitative analysis of the Au coverage, extracted from optical microscopy images (outlined in the selected region), is presented in Figure S9c. The Au coverage decreased from nearly 100% at 0 min to ~95% after 2 min exposure, and further reduced to below 50% following 12 min exposure, highlighting the time-dependent efficiency of iodine vapor etching.

**8. Density Functional Theory Calculation (DFT)**

To evaluate the adsorption of MoS_2_ on the continuous and a partially etched Au, we performed density functional tight binding (DFTB) method with DFTB+ software package^[1]^ and GFN1-xTB method^[2]^. The top view and the side view of the MoS_2_ and Au surface is shown as figure S7. To mimic the partially etched state, we create a trench-like Au surface. The adsorption energy of MoS_2_ on Au is calculated using the following equation^[3]^:

E_adsorption_ = E_system_ – (E_MoS2_ + E_Au_)

where E_{adsorption} is the adsorption energy, E_{system} is the total energy of the MoS_2_ and Au substrate system, E_{MoS_2_}, and E_{Au} is the energy of the Au substrate. For the continuous Au substrate, the adsorption energy per area is about -1.43 Jm^{-2}. As the trench size increases, mimicking the size of the etching state increases, the value of adsorption energy decreases. Here the value of adsorption energies is negative, indicating that the MoS_2_ is favored to adsorb on the Au surface. As the trench size increases, the adsorption energy become less negative, indicating that the binding between MoS_2_ and Au surface become smaller, which is consistent with the experimental observations of progressive PL restoration.

**Figure S10.** MoS_2_-Au heterostructure (both top and side view) and the computed adsorption energy (in J/m^2^) as a function of different trench sizes.

**Table S1.** Density functional tight binding (DFTB) output results.

| **Trench** | **Au_MoS2** | **Au** | **MoS2** | **E_adsorption** | **Trench Size (Å)** | **Area (Å)^2** | **Energy per area (eV/Å^2)** | **Energy per area (J/m^2)** |
| --- | --- | --- | --- | --- | --- | --- | --- | --- |
| 0 | -53444.7023 | -39270.0768 | -14128.2266 | -46.3989 | 0 | 518.5382794 | -0.089480183 | -1.433472528 |
| 1 | -50154.4267 | -35981.2106 | -14128.2266 | -44.9895 | 5.76752 | 518.5382794 | -0.086762158 | -1.389929767 |
| 2 | -46874.1333 | -32704.5212 | -14128.2266 | -41.3855 | 8.6345 | 518.5382794 | -0.079811851 | -1.278585856 |
| 3 | -43596.7513 | -29430.922 | -14128.2266 | -37.6027 | 11.535 | 518.5382794 | -0.072516729 | -1.161718002 |
| 4 | -40320.4029 | -26158.6707 | -14128.2266 | -33.5056 | 14.4428 | 518.5382794 | -0.06461548 | -1.035139995 |
| 5 | -37044.2034 | -22886.4073 | -14128.2266 | -29.5695 | 17.3025 | 518.5382794 | -0.05702472 | -0.913536009 |
| 6 | -33767.5319 | -19613.4151 | -14128.2266 | -25.8902 | 20.1669 | 518.5382794 | -0.049929197 | -0.799865739 |
| 7 | -30491.0002 | -16340.4652 | -14128.2266 | -22.3084 | 23.0701 | 518.5382794 | -0.043021703 | -0.689207687 |
| 8 | -27214.9382 | -13068.4429 | -14128.2266 | -18.2687 | 25.934 | 518.5382794 | -0.03523115 | -0.564403026 |
| 9 | -23938.9105 | -9796.4937 | -14128.2266 | -14.1902 | 28.8376 | 518.5382794 | -0.027365771 | -0.438399657 |
| 10 | -20663.2553 | -6524.3535 | -14128.2266 | -10.6752 | 31.7439 | 518.5382794 | -0.020587101 | -0.32980536 |
| 11 | -17388.4396 | -3253.3 | -14128.2266 | -6.913 | 34.60509877 | 518.5382794 | -0.013331706 | -0.213573934 |

Density functional tight binding (DFTB) calculations were carried out to quantify the effect of partial Au removal on the interfacial coupling strength with MoS_2_ (Fig. S10). Two representative interfacial configurations were considered: (i) MoS_2_ supported on continuous Au and (ii) MoS_2_ on trench-like Au, mimicking the partially etched morphology observed during iodine vapor exposure. For the continuous Au substrate, the calculated adsorption energy was −1.43 J m^-2^, reflecting strong interfacial interaction that is consistent with the previous report. In contrast, as the trench size increased, the adsorption energy became progressively less negative, reaching -0.20 J m^-2^ for a trench width of ~35 Å. This substantial reduction in binding energy confirms the weakening of the MoS_2_-Au coupling as the Au support becomes discontinuous. These theoretical results align closely with the experimental observation of PL recovery and blue-shifted excitonic emission, providing direct evidence that the biphasic etching process effectively modulates the interfacial energetics by reducing metal-2D coupling.

**9. Raman Mapping of Polymer Transferred MoS_2_ (PT-MoS_2_)**

**Figure S11.** (a) High-magnification optical image of polymer-transferred MoS_2_ (PT-MoS_2_); the blue square outlines the region selected for Raman mapping. (b, c) Corresponding Raman intensity maps of the E_2g_ and A_1g_ modes, respectively. The mapping reveals structural discontinuities and defect-related inhomogeneities induced during the polymer transfer process.

**10. Power-Dependent Photocurrent Analysis of MoS_2_-Based Photodetectors**

**Figure S12.** The power-dependent photocurrent response of biphasic etched MoS_2_ (BE-MoS_2_) and polymer transferred MoS_2_ (PT-MoS_2_).

**Table S2.** Literature comparison on previously reported MoS_2_ photodetector performances (1L = monolayer; 2L = Bilayer; FL = Few layer)

| **Sample** | **Synthesis Method** | **Transfer** | **Response time (s)** | **Ref.** |
| --- | --- | --- | --- | --- |
| MoS_2_ – 1L | Mechanical Exfoliation | None | 4 | 4 |
| MoS_2_ – 2L | Sputter | None | 0.3 | 5 |
| MoS_2_ – 1L | Exfoliation | PVA/PMMA | 0.23 | 6 |
| MoS_2_ – 1L | CVD | PMMA | 31 | 7 |
| MoS_2_ – 1L | CVD | PMMA | 0.35 | 8 |
| MoS_2_ – 2L | CVD | PMMA | 4.94 | 9 |
| MoS_2_ – FL | Bath Sonication | Xanthan gum | 1 | 10 |
| MoS_2_ – FL | Bath Sonication | PI | 3 | 11 |
| MoS_2_ – 1L | MME | None | 0.128 | This work |

**Table S3.** Literature comparison on previously reported polymer-free methods

| 2D Material | Synthesis Method | Transfer Method | Polymer free | Transfer free | Ref. |
| --- | --- | --- | --- | --- | --- |
| Graphene | CVD | PDMS Stamping | Yes | No | 12 |
| Graphene | CVD | WTP | yes | No | 13 |
| Graphene | CVD | Wet transfer | Yes | No | 14 |
| Graphene | CVD | Wet transfer | Yes | No | 15 |
| Graphene | Mechanical exfoliation | Dry transfer | Yes | No | 16 |
| MoS_2_ | CVD | SiO_2_ etching | Yes | No | 17 |
| MoS_2_ | CVD | Wet transfer | Yes | No | 18 |
| MoS_2_ | CVD | Wet transfer | Yes | No | 19 |
| MoS_2_ | CVD | Ice Transfer | Yes | No | 20 |
| MoS_2_ | Mechanical exfoliation | Tape Stamp | Yes | Yes | 21 |
| hBN | Mechanical exfoliation | Laser-assisted transfer | Yes | No | 22 |
| MoS_2_ | Metal assisted mechanical exfoliation (MME) | Biphasic etching | Yes | Yes | This work |

**Reference**

1. Spiegelman, F.; Tarrat, N.; Cuny, J.; Dontot, L.; Posenitskiy, E.; Martí, C.; Simon, A.; Rapacioli, M. Density-functional tight-binding: basic concepts and applications to molecules and clusters. *Advances in physics: X* **2020**, *5* (1), 1710252.

2. Grimme, S.; Bannwarth, C.; Shushkov, P. A robust and accurate tight-binding quantum chemical method for structures, vibrational frequencies, and noncovalent interactions of large molecular systems parametrized for all spd-block elements (Z= 1–86). *Journal of chemical theory and computation* **2017**, *13* (5), 1989-2009.

3. Bahamon, D.; Khalil, M.; Belabbes, A.; Alwahedi, Y.; Vega, L. F.; Polychronopoulou, K. A DFT study of the adsorption energy and electronic interactions of the SO 2 molecule on a CoP hydrotreating catalyst. *RSC advances* **2021**, *11* (5), 2947-2957.

4. Lopez-Sanchez, O.; Lembke, D.; Kayci, M.; Radenovic, A.; Kis, A. Ultrasensitive photodetectors based on monolayer MoS2. *Nature nanotechnology* **2013**, *8* (7), 497-501.

5. Ling, Z.; Yang, R.; Chai, J.; Wang, S.; Leong, W.; Tong, Y.; Lei, D.; Zhou, Q.; Gong, X.; Chi, D. Large-scale two-dimensional MoS2 photodetectors by magnetron sputtering. *Optics express* **2015**, *23* (10), 13580-13586.

6. Vu, Q. A.; Lee, J. H.; Nguyen, V. L.; Shin, Y. S.; Lim, S. C.; Lee, K.; Heo, J.; Park, S.; Kim, K.; Lee, Y. H. Tuning carrier tunneling in van der Waals heterostructures for ultrahigh detectivity. *Nano letters* **2017**, *17* (1), 453-459.

7. Liu, H.; Gao, F.; Hu, Y.; Zhang, J.; Wang, L.; Feng, W.; Hou, J.; Hu, P. Enhanced photoresponse of monolayer MoS2 through hybridization with carbon quantum dots as efficient photosensitizer. *2D Materials* **2019**, *6* (3), 035025.

8. Li, Y.; Li, L.; Li, S.; Sun, J.; Fang, Y.; Deng, T. Highly sensitive photodetectors based on monolayer MoS2 field-effect transistors. *ACS omega* **2022**, *7* (16), 13615-13621.

9. Wang, W.; Klots, A.; Prasai, D.; Yang, Y.; Bolotin, K. I.; Valentine, J. Hot electron-based near-infrared photodetection using bilayer MoS2. *Nano letters* **2015**, *15* (11), 7440-7444.

10. Alzakia, F. I.; Jonhson, W.; Ding, J.; Tan, S. C. Ultrafast exfoliation of 2D materials by solvent activation and one-step fabrication of all-2D-material photodetectors by electrohydrodynamic printing. *ACS applied materials & interfaces* **2020**, *12* (25), 28840-28851.

11. Kuo, L.; Sangwan, V. K.; Rangnekar, S. V.; Chu, T. C.; Lam, D.; Zhu, Z.; Richter, L. J.; Li, R.; Szydłowska, B. M.; Downing, J. R. All‐printed ultrahigh‐responsivity MoS2 nanosheet photodetectors enabled by megasonic exfoliation. *Advanced Materials* **2022**, *34* (34), 2203772.

12. Fujihara, M.; Ogawa, S.; Yoshimura, S.; Inoue, R.; Maniwa, Y.; Taniguchi, T.; Watanabe, K.; Shinohara, H.; Miyata, Y. Clean and polymer-free transfer of CVD-grown graphene films on hexagonal boron nitride substrates. Japanese Journal of Applied Physics 2017, 56 (5), 055102.

13. Zhang, X.; Xu, C.; Zou, Z.; Wu, Z.; Yin, S.; Zhang, Z.; Liu, J.; Xia, Y.; Lin, C.-T.; Zhao, P. A scalable polymer-free method for transferring graphene onto arbitrary surfaces. Carbon 2020, 161, 479-485.

14. Zhang, G.; Guell, A. G.; Kirkman, P. M.; Lazenby, R. A.; Miller, T. S.; Unwin, P. R. Versatile polymer-free graphene transfer method and applications. ACS applied materials & interfaces 2016, 8 (12), 8008-8016.

15. Lin, W.-H.; Chen, T.-H.; Chang, J.-K.; Taur, J.-I.; Lo, Y.-Y.; Lee, W.-L.; Chang, C.-S.; Su, W.-B.; Wu, C.-I. A direct and polymer-free method for transferring graphene grown by chemical vapor deposition to any substrate. ACS nano 2014, 8 (2), 1784-1791.

16. Wang, W.; Tainton, G. R.; Clark, N. J.; McHugh, J. G.; Li, X.; Sullivan-Allsop, S.; Hopkinson, D. G.; Cicvárek, O.; Selles, F.; Zhang, R. Atomic Imaging of 2D Transition Metal Diiodides. ACS nano 2026.

17. Yang, J.; Choi, M. K.; Sheng, Y.; Jung, J.; Bustillo, K.; Chen, T.; Lee, S.-W.; Ercius, P.; Kim, J. H.; Warner, J. H. MoS2 liquid cell electron microscopy through clean and fast polymer-free MoS2 transfer. Nano letters 2019, 19 (3), 1788-1795.

18. Borrazás, C.; Aversa, M.; Bragas, A.; Grinblat, G. MÉTODO DE TRANSFERENCIA HÚMEDA LIBRE DE POLÍMEROS DE MATERIALES VAN DER WAALS. In ANALES AFA, 2025; Vol. 36, pp 90-94.

19. Hong, M.; Yang, P.; Zhou, X.; Zhao, S.; Xie, C.; Shi, J.; Zhang, Z.; Fu, Q.; Zhang, Y. Decoupling the interaction between wet‐transferred MoS2 and graphite substrate by an interfacial water layer. Advanced Materials Interfaces 2018, 5 (21), 1800641.

20. Liu, H.; Thi, Q. H.; Man, P.; Chen, X.; Chen, T.; Wong, L. W.; Jiang, S.; Huang, L.; Yang, T.; Leung, K. H. Controlled adhesion of ice—toward ultraclean 2D materials. Advanced Materials 2023, 35 (14), 2210503.

21. Lee, M.; Kim, C.; Kwon, S. Y.; Lee, K.; Kwak, G.; Lim, H.; Seol, J. H. Residue‐free fabrication of 2D materials using van der Waals interactions. Advanced Materials 2025, 37 (21), 2418669.

22. Goodfriend, N. T.; Mirza, I.; Bulgakov, A. V.; Campbell, E. E.; Bulgakova, N. M. Laser-induced gas-phase transfer and direct stamping of nanomaterials: comparison of nanosecond and femtosecond pulses. Materials Research Express 2025, 12 (6), 065004.
